# Effects of tesamorelin on hepatic transcriptomic signatures in HIV-associated NAFLD

**DOI:** 10.1172/jci.insight.140134

**Published:** 2020-08-20

**Authors:** Lindsay T. Fourman, James M. Billingsley, George Agyapong, Shannan J. Ho Sui, Meghan N. Feldpausch, Julia Purdy, Isabel Zheng, Chelsea S. Pan, Kathleen E. Corey, Martin Torriani, David E. Kleiner, Colleen M. Hadigan, Takara L. Stanley, Raymond T. Chung, Steven K. Grinspoon

**Affiliations:** 1 Metabolism Unit, Massachusetts General Hospital and Harvard Medical School, Boston, Massachusetts, USA.; 2 Harvard Chan Bioinformatics Core, Department of Biostatistics, Harvard T.H. Chan School of Public Health, Boston, Massachusetts, USA.; 3 Liver Center, Digestive Healthcare Center, Massachusetts General Hospital and Harvard Medical School, Boston, Massachusetts, USA.; 4 National Institute of Allergy and Infectious Diseases, NIH, Bethesda, Maryland, USA.; 5 Department of Radiology, Massachusetts General Hospital and Harvard Medical School, Boston, Massachusetts, USA.; 6 Laboratory of Pathology, Center for Cancer Research, National Cancer Institute, NIH, Bethesda, Maryland, USA.

**Keywords:** AIDS/HIV, Hepatology, Fibrosis, growth factors

## Abstract

Nonalcoholic fatty liver disease (NAFLD) is a common comorbidity among people living with HIV that has a more aggressive course than NAFLD among the general population. In a recent randomized placebo-controlled trial, we demonstrated that the growth hormone–releasing hormone analog tesamorelin reduced liver fat and prevented fibrosis progression in HIV-associated NAFLD over 1 year. As such, tesamorelin is the first strategy that has shown to be effective against NAFLD among the population with HIV. The current study leveraged paired liver biopsy specimens from this trial to identify hepatic gene pathways that are differentially modulated by tesamorelin versus placebo. Using gene set enrichment analysis, we found that tesamorelin increased hepatic expression of hallmark gene sets involved in oxidative phosphorylation and decreased hepatic expression of gene sets contributing to inflammation, tissue repair, and cell division. Tesamorelin also reciprocally up- and downregulated curated gene sets associated with favorable and poor hepatocellular carcinoma prognosis, respectively. Notably, among tesamorelin-treated participants, these changes in hepatic expression correlated with improved fibrosis-related gene score. Our findings inform our knowledge of the biology of pulsatile growth hormone action and provide a mechanistic basis for the observed clinical effects of tesamorelin on the liver.

## Abstract 2

In individuals with HIV and nonalcoholic fatty liver disease, tesamorelin upregulated hepatic expression of genes related to oxidative phosphorylation and downregulated genes related to inflammation and liver fibrosis.

## Graphical abstract

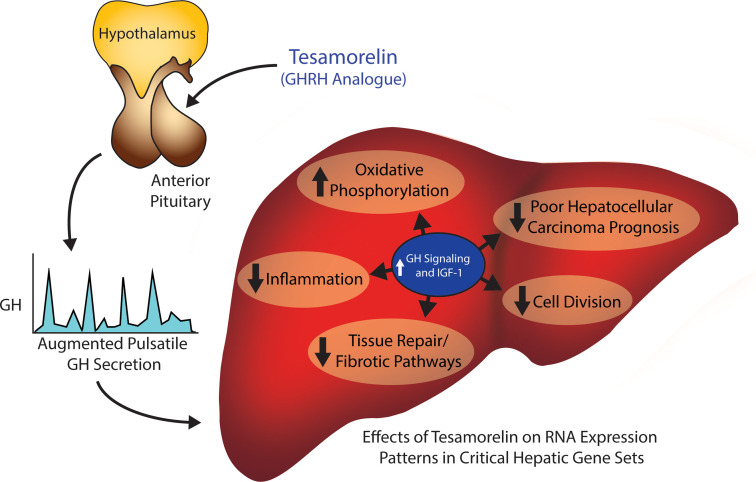

## Introduction

Nonalcoholic fatty liver disease (NAFLD) has become a leading cause of liver disease worldwide. NAFLD encompasses a broad spectrum of disease that ranges histologically from simple steatosis to steatohepatitis (NASH) to fibrosis and may ultimately progress to cirrhosis or hepatocellular carcinoma (HCC). NAFLD is a major comorbidity among people living with HIV (PLWH), with over one-third of individuals affected (1). Compared with the general population, PLWH have a more aggressive disease course that is characterized by a greater prevalence of NASH and fibrosis as well as an accelerated rate of fibrosis progression (2).

Despite the heightened burden of NAFLD in HIV, approved pharmacologic interventions to treat this condition in HIV are lacking. Tesamorelin, a hypothalamic growth hormone–releasing hormone (GHRH) analog that augments endogenous pulsatile growth hormone (GH) and downstream insulin-like growth factor-1 (IGF-1) secretion, is FDA-approved to reduce visceral adiposity in HIV. In a recent randomized placebo-controlled trial, we investigated for the first time to our knowledge the effects of this agent on liver fat and histology among individuals with HIV-associated NAFLD. Through this work, we found that tesamorelin significantly reduced liver fat and prevented fibrosis progression over 1 year (3).

While high-throughput gene expression technologies have been widely used to elucidate key pathways predictive of NAFLD course among the general population, few studies have examined the effects of potential therapeutic strategies on hepatic transcriptomic signatures in any patient group. In the current study, we leveraged paired liver biopsy specimens from our recent clinical trial to investigate differential changes in hepatic gene expression by tesamorelin versus placebo over 1 year. Through this analysis, we identified novel biological pathways changing in tesamorelin versus placebo that may underlie the phenotypic changes that we observed in our clinical trial. Our findings expand upon the known effects of tesamorelin on hepatic pathophysiology, using a transcriptomic approach to identify effects on gene signatures encompassing major inflammatory and fibrotic pathways and increasing our understanding of the physiologic effects of augmented pulsatile GH secretion on the liver transcriptome.

## Results

### Characteristics of study participants

Demographic and clinical characteristics were similar between treatment groups. Data for individual treatment groups are shown in Table 1. In the overall sample, participants (53 ± 7 years old, 77% male) had chronic HIV infection (17 ± 9 years) with excellent virologic control. All subjects were on stable antiretroviral therapy (ART), with 64% receiving integrase inhibitor–based regimens. Baseline hepatic fat fraction in our cohort was 14% ± 9%. Moreover, 31% and 41% of participants had histologically defined NASH and fibrosis, respectively. Over the study period, changes in body weight, dietary intake, and other relevant clinical factors were not found to differ between treatment groups, as has been previously described (3). Baseline characteristics of the participants included in this analysis with transcriptomic data on paired biopsy specimens did not differ substantially from that of the participants in the larger parent trial (see Supplemental Table 1; supplemental material available online with this article; https://doi.org/10.1172/jci.insight.140134DS1).

### Effects of tesamorelin on expression of hallmark gene sets

**Figure 1:**
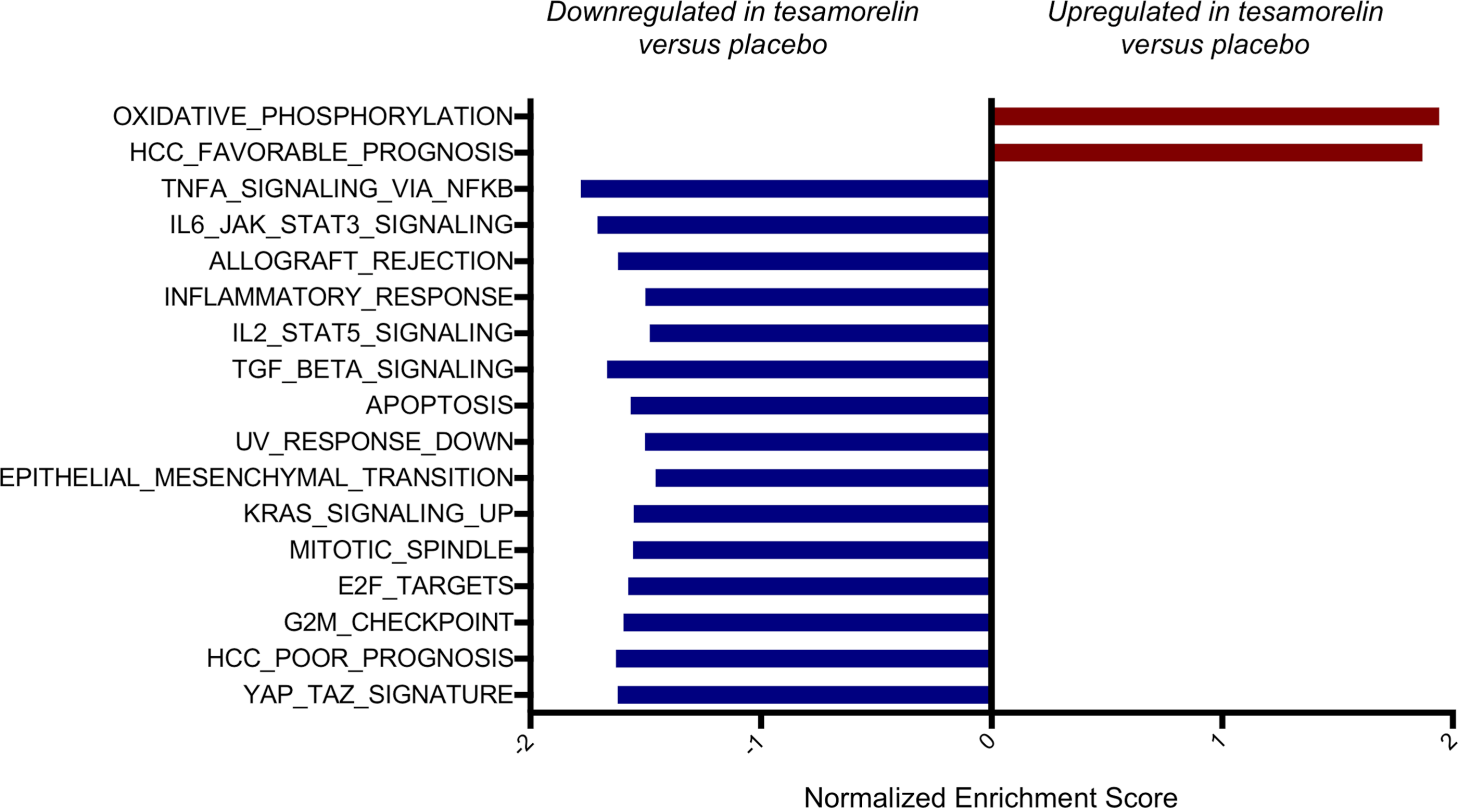
Hepatic gene expression pathways were differentially modulated by tesamorelin versus placebo. A bar graph depicts the normalized enrichment score for hallmark and curated gene sets whose hepatic expression was differentially regulated by tesamorelin versus placebo. Two gene sets pertaining to oxidative phosphorylation and favorable prognosis of hepatocellular carcinoma (HCC) were found to be upregulated in tesamorelin- versus placebo-treated participants. Fifteen gene sets related to inflammation, tissue repair, cell turnover, and poor prognosis of HCC were found to be downregulated in tesamorelin- versus placebo-treated participants. For each pathway shown, the difference between groups had FDR *q* value less than 0.05.

We first performed an unbiased analysis to identify hepatic biological pathways differentially modulated by tesamorelin versus placebo using gene set enrichment analysis (GSEA), which determines whether a priori–defined sets of genes defining distinct biological pathways are differentially expressed between 2 conditions. In pathways that are significantly enriched at either the top or bottom of a transcriptome ranked by differential expression, the leading edge genes are the subset of genes that contribute most to the enrichment signal. For target gene sets, we queried the Molecular Signatures Database (MSigDB) hallmark gene sets (4), which consist of 50 gene sets that represent well-defined biological states or processes (5). Using this approach, we found 14 hallmark gene sets to be differentially regulated between treatment groups with false discovery rate (FDR) *q* value less than 0.05 (Table 2 and Figure 1). In this regard, a gene set encoding oxidative phosphorylation proteins was upregulated by tesamorelin versus placebo. Moreover, 13 gene sets pertaining to inflammation, tissue repair, and cell division were downregulated in the tesamorelin group relative to the placebo group. There was minimal overlap of leading edge genes between the differentially modulated gene sets (Supplemental Figure 1).

#### Cell metabolism.

**Figure 2:**
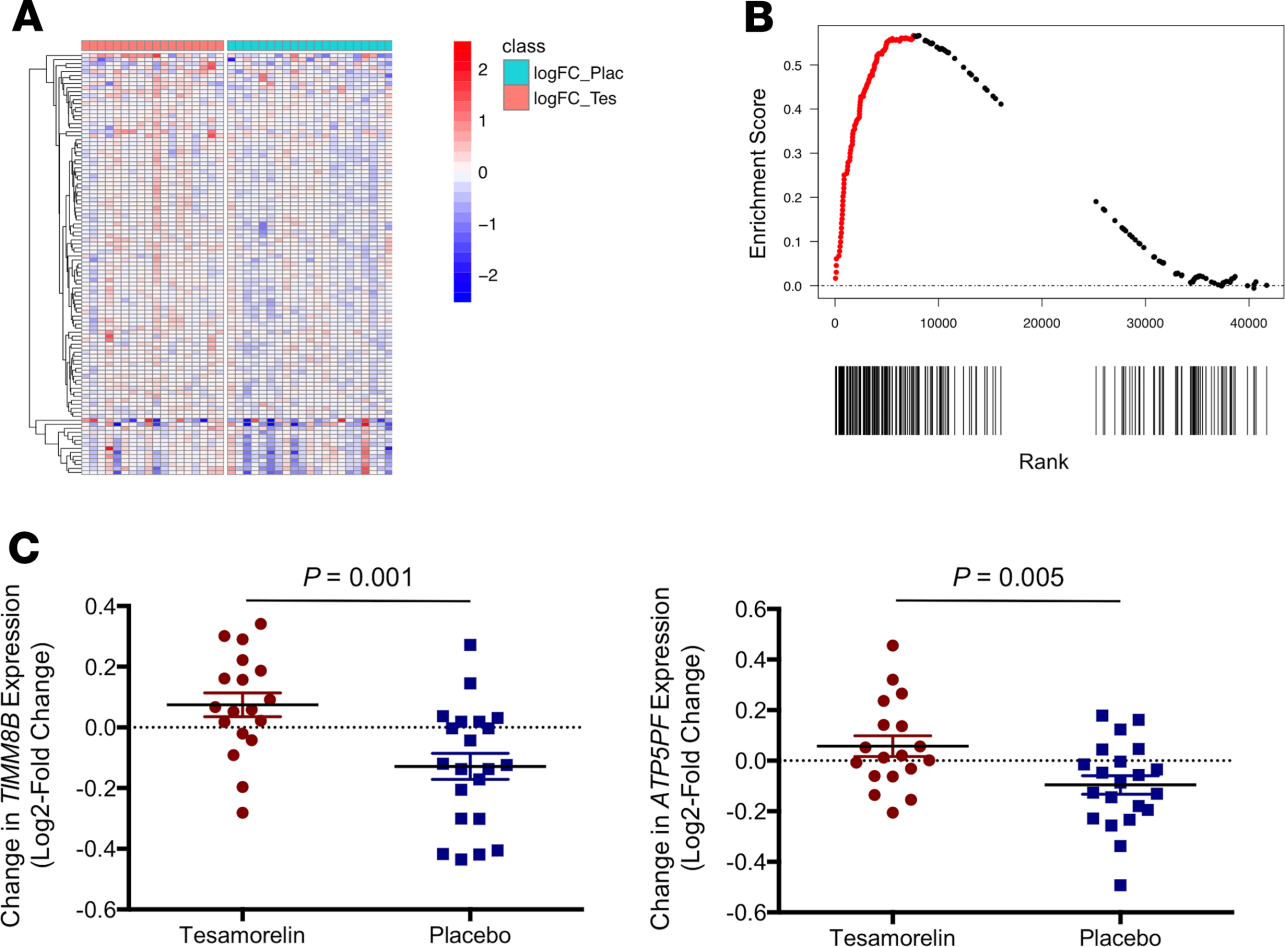
Tesamorelin mediated hepatic upregulation of the oxidative phosphorylation hallmark gene set. (**A**) Heatmap of log_2_ fold changes in hepatic gene expression for the hallmark OXIDATIVE_PHOSPHORYLATION gene set. Columns correspond to individual participants; rows represent log_2_ fold change of individual leading edge genes. There was overall upregulation of oxidative phosphorylation genes among tesamorelin-treated participants (left) and downregulation of oxidative phosphorylation genes among placebo-treated participants (right). (**B**) GSEA enrichment plot for the hallmark OXIDATIVE_PHOSPHORYLATION gene set. The position of each gene in the gene set in the ranked transcriptome is indicated by a black bar below the plot. Clustering of genes to the left on the ranked list indicates greater upregulation in tesamorelin relative to placebo. Genes corresponding to the leading edge are shown in red. (**C**) Dot plots of changes in hepatic gene expression for select genes within the OXIDATIVE_PHOSPHORYLATION gene set. Compared with placebo-treated patients (*N* = 21), tesamorelin-treated participants (*N* = 18) exhibited hepatic upregulation of *TIMM8B*, which is responsible for guiding membrane-spanning proteins into the mitochondrial inner membrane. Individuals assigned to tesamorelin also demonstrated enhanced expression of *ATP5PF*, which encodes a subunit of the mitochondrial ATP synthase (complex V). *P* values were corrected for multiple testing and calculated using negative binomial generalized linear models. Error bars correspond to mean and standard error of the mean.

**Figure 3:**
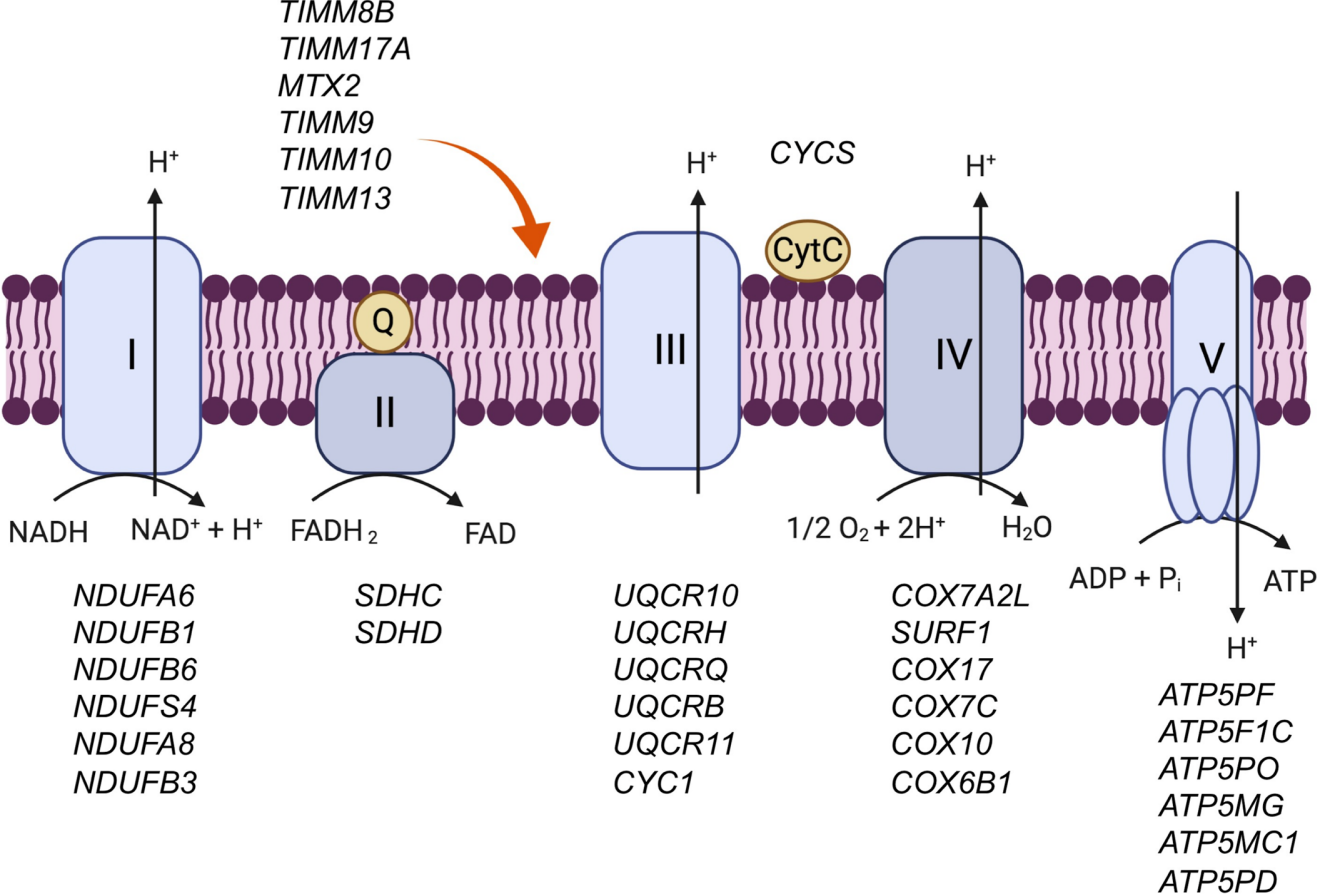
Oxidative phosphorylation genes upregulated by tesamorelin included genes involved in the electron transport chain. Select genes within the leading edge of the OXIDATIVE_PHOSPHORYLATION gene set are listed beside the complex to which they correspond. Tesamorelin was found to modulate genes responsible for the structure and function of all 5 complexes within the electron transport chain.

The OXIDATIVE_PHOSPHORYLATION hallmark gene set was differentially regulated between tesamorelin and placebo (normalized enrichment score [NES] = 1.94, FDR *q* value = 0.0005). In particular, we found that genes involved in oxidative phosphorylation were globally upregulated among tesamorelin-treated participants and downregulated among placebo-treated participants (Figure 1 and Figure 2). A large proportion of leading edge genes within this gene set encoded subunits of the electron transport chain (Figure 3). Examples included *NDUFA6* and *NDUFB1* of complex I, *SDHC* and *SDHD* of complex II, *UQCR10* and *UQCRH* of complex III, *COX7A2L* and *COX17* of complex IV, and *ATP5PF* and *ATP5F1C* of complex V. Relatedly, other genes within the leading edge supported the electron transport chain by participating in the import and insertion of transmembrane proteins into the mitochondrial inner membrane, such as *TIMM8B* and *TIMM9*. Last, a subset of leading edge genes encoded enzymes involved in cell catabolism, including *ECI1* and *ECHS1* of the fatty acid β-oxidation pathway and *FH* and *IDH3B* of the tricarboxylic acid cycle. Among all participants, changes in expression of oxidative phosphorylation genes related to changes in IGF-1 transcription (*r* = 0.35, *P* = 0.03), change in serum IGF-1 (*r* = 0.45, *P* = 0.005), and change in visceral fat content (*r* = –0.42, *P* = 0.008) but not to change in fasting glucose (*r* = 0.06, *P* = 0.7).

#### Inflammation.

**Figure 4:**
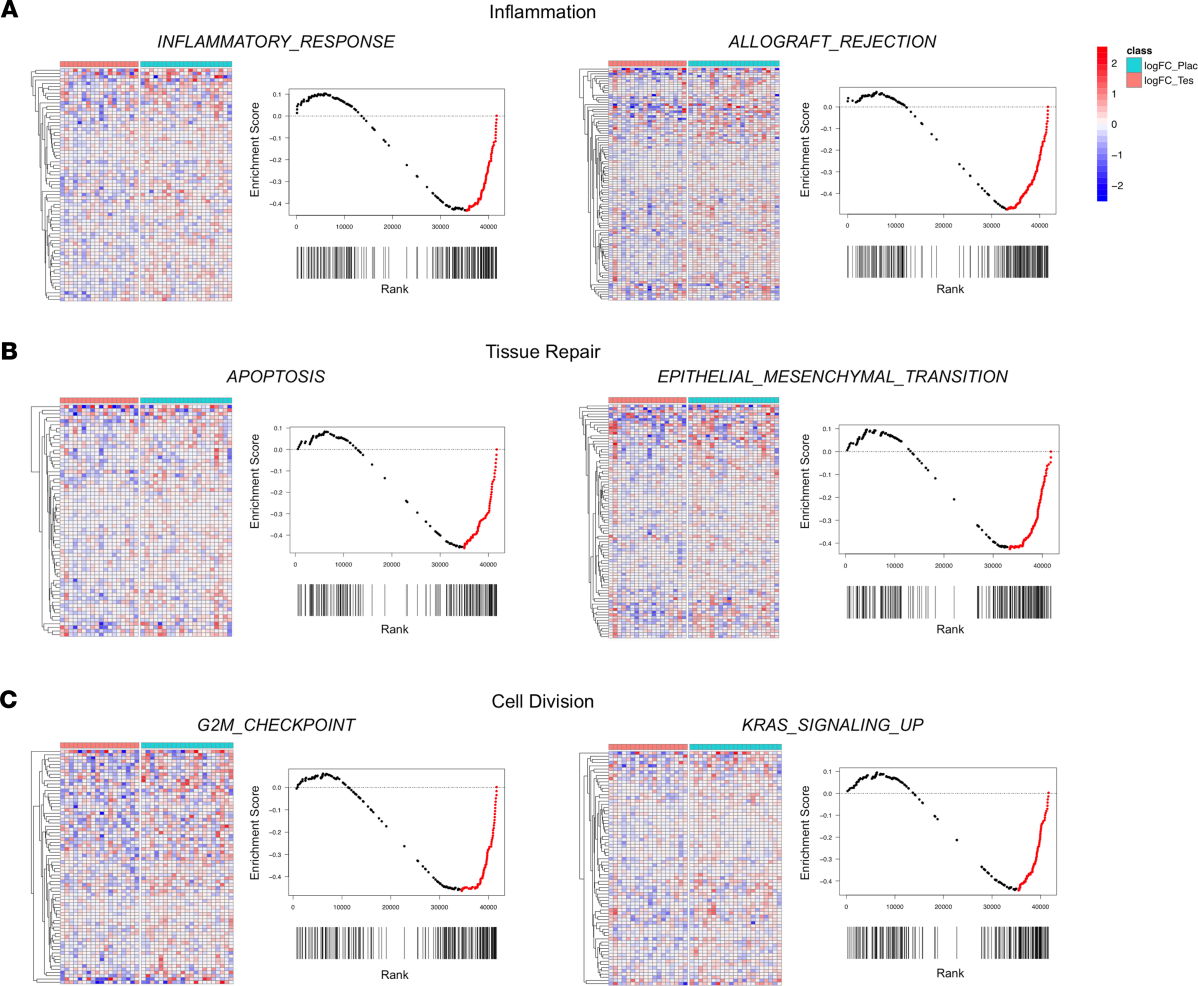
Tesamorelin led to hepatic downregulation of hallmark gene sets involved in inflammation, tissue repair, and cell division. Heatmaps and enrichment plots demonstrate differential changes in hepatic expression of select gene sets involved in (**A**) inflammation, (**B**) tissue repair, and (**C**) cell turnover by treatment status. For the heatmaps, columns correspond to individual participants, whereas rows represent log_2_ fold change of individual leading edge genes. Overall, these pathways were downregulated among tesamorelin-treated participants (left) and upregulated among placebo-treated participants (right). Enrichment plots similarly show that genes within the featured gene sets were overrepresented at the bottom of the entire ranked list. Genes corresponding to the leading edge are shown in red.

Five hallmark gene sets pertaining to inflammation were differentially regulated by tesamorelin versus placebo: TNFA_SIGNALING_VIA_NFKB (NES = –1.78, FDR *q* value = 0.01), IL6_JAK_STAT3_SIGNALING (NES = –1.71, FDR *q* value = 0.02), ALLOGRAFT_REJECTION (NES = –1.62, FDR *q* value = 0.03), INFLAMMATORY_RESPONSE (NES = –1.50, FDR *q* value = 0.03), and IL2_STAT5_SIGNALING (NES = –1.48, FDR *q* value = 0.03). Overall, all 5 gene sets were downregulated in the tesamorelin group and upregulated in the placebo group (Figure 4). Collectively, the leading edges of these gene sets comprised genes involved in the function of the innate and adaptive immune response. These included genes encoding immune cell components, such as *CD8B*, *CD3D*, and *CD4*, which are involved in T cell receptor activation, and *HLA-DQA1*, which is involved in antigen presentation. In addition, genes needed for recruitment and maintenance of immune cells at sites of tissue injury were also prominent within the leading edges of the inflammatory gene sets. Examples were *CCL5* and *CCL20* encoding cytokines chemotactic for diverse immune cell types, *ICAM1* encoding a cell surface adhesion molecule important for leukocyte transendothelial migration, and *IL15RA* mediating survival of T cells and natural killer cells.

#### Tissue repair.

Four hallmark gene sets related to tissue repair were differentially regulated by tesamorelin versus placebo: TGF_BETA_SIGNALING (NES = –1.67, FDR *q* value = 0.03), APOPTOSIS (NES = –1.56, FDR *q* value = 0.03), UV_RESPONSE_DN (NES = –1.50, FDR *q* value = 0.03), and EPITHELIAL_MESENCHYMAL_TRANSITION (NES = –1.46, FDR *q* value = 0.04). In general, all 4 gene sets were downregulated among tesamorelin-treated participants, whereas they were upregulated among the placebo-treated arm (Figure 4). Within the leading edges of these gene sets were genes important for extracellular matrix (ECM) remodeling, including *BGN*, *SERPINH1*, and *COL1A1* responsible for collagen biosynthesis and assembly, *MMP14* and *MMP2* encoding metalloproteinases that mediate ECM degradation, and *TIMP1* encoding a metalloproteinase inhibitor. Also included within the leading edges of these gene sets were genes that contribute to epithelial-mesenchymal transition (EMT), such as *DAB2*, a critical switch required for EMT, along with *VIM* and *CDH2* encoding key mesenchymal markers. Last, genes that contribute to apoptosis, such as *PMAIP1*, *CASP8*, and *BCL10*, and genes that are important for transforming growth factor–β (TGF-β) signaling, such as *RHOA*, *TGFB1*, and *TGFBR1*, were included within the leading edges.

#### Cell division.

Four hallmark gene sets involved in cell division were differentially modulated by tesamorelin versus placebo: G2M_CHECKPOINT (NES = –1.60, FDR *q* value = 0.03), E2F_TARGETS (NES = –1.57, FDR *q* value = 0.03), MITOTIC_SPINDLE (NES = –1.56, FDR *q* value = 0.03), and KRAS_SIGNALING_UP (NES = –1.55, FDR *q* value = 0.02). Overall, these gene sets were downregulated among tesamorelin-treated participants and upregulated among placebo-treated participants (Figure 4). The leading edges of these gene sets include genes responsible for DNA replication, including the minichromosome maintenance genes *MCM6* and *MCM2* and the DNA polymerase *POLD1*. Also prominent within these leading edges were genes important for mitotic chromosomal segregation, such as *CENPJ* and *CEP192* involved in centrosome function, *CLASP1* and *NUMA1* involved in mitotic spindle formation, and *STAG1* and *SMC1A* required for cohesion of sister chromatids. Last, genes that encode positive cell cycle regulators, including *CCNB2*, *CDK1*, *E2F2*, and *E2F1*, as well as the marker of cell proliferation *MKI67*, were also represented among the leading edges of these gene sets, indicating their relative downregulation among the tesamorelin group.

### Effects of tesamorelin on curated gene sets prognostic of HCC

**Figure 5:**
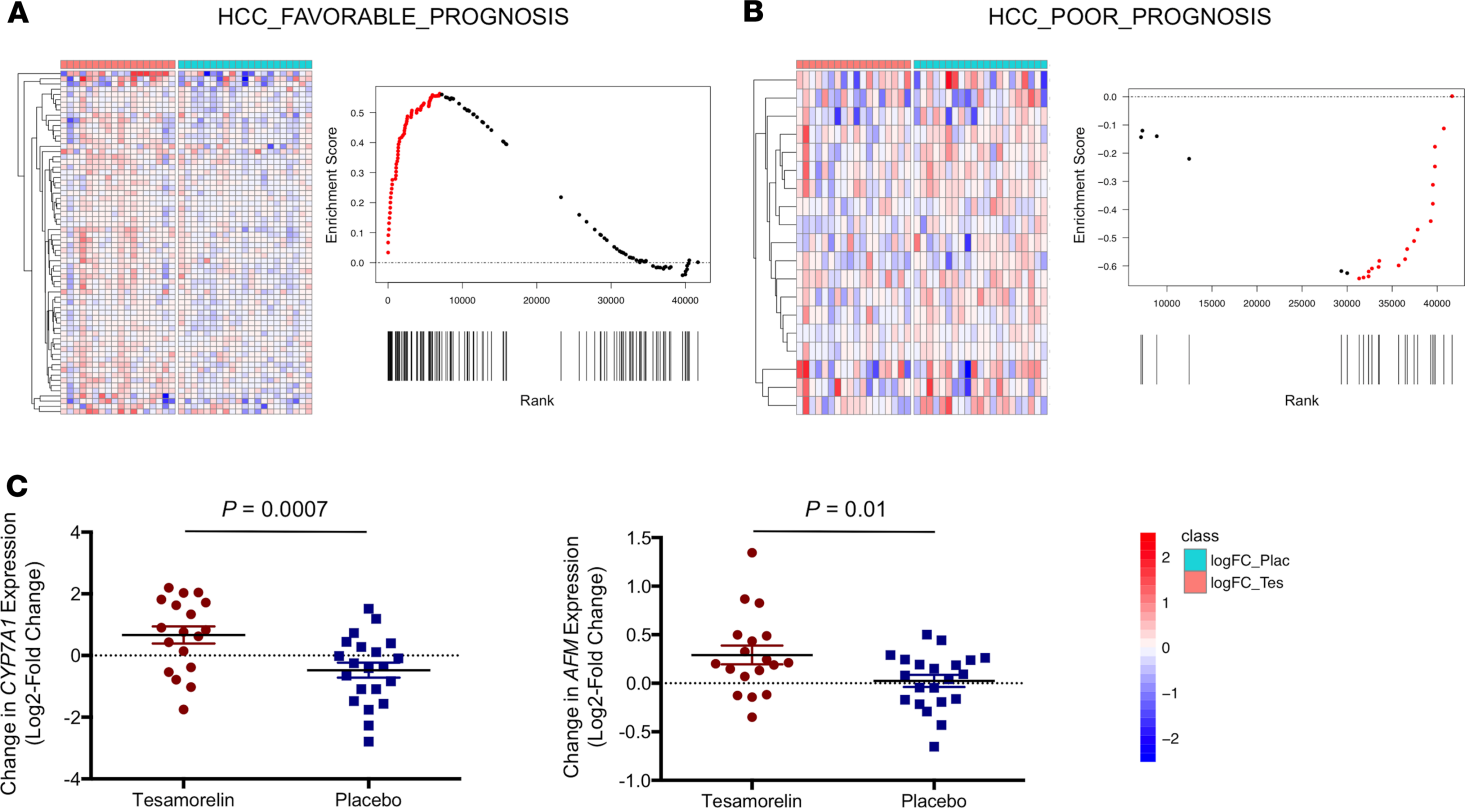
Tesamorelin shifted hepatic gene expression toward a profile associated with a more favorable HCC prognosis. (**A**) A heatmap and an enrichment plot are shown, depicting changes in hepatic expression of a curated gene set associated with favorable HCC prognosis. In the heatmap, columns correspond to individual participants, whereas rows represent log_2_ fold change of individual leading edge genes. Genes predictive of a favorable HCC prognosis were overall upregulated among tesamorelin-treated participants (left) and downregulated among placebo-treated participants (right). The enrichment plot likewise demonstrates an overrepresentation of genes associated with favorable prognosis at the top of the entire ranked list. Genes corresponding to the leading edge are shown in red. (**B**) A heatmap and an enrichment plot demonstrate changes in hepatic expression of a curated gene set associated with poor HCC prognosis. In contrast to our findings with regard to the favorable prognosis gene set, genes linked to a poor HCC prognosis were reciprocally downregulated and upregulated in the tesamorelin and placebo groups, respectively. Furthermore, per the enrichment plot shown, genes within the poor prognosis gene set were overrepresented at the bottom of the entire ranked list. (**C**) Dot plots of changes in hepatic gene expression for select genes within the favorable prognosis gene set are shown. Compared with placebo-treated patients (*N* = 21), tesamorelin-treated participants (*N* = 18) were found to have hepatic upregulation of *CYP7A1* encoding a critical regulatory enzyme in bile acid biosynthesis and cholesterol homeostasis and *AFM* encoding a member of the albumin gene family responsible for protein transport. *P* values were corrected for multiple testing and calculated using negative binomial generalized linear models. Error bars correspond to mean and standard error of the mean.

We next examined curated gene sets that were prognostic of HCC to test the hypothesis that tesamorelin would confer a favorable gene expression profile in this regard. We found that tesamorelin relative to placebo led to upregulation of genes associated with favorable HCC prognosis (NES = 1.87, FDR *q* value = 0.0003) (Table 3 and Figure 5). Contained within the leading edge of this gene set were genes responsible for hepatic homeostatic functions. Examples included *CYP7A1*, *SLC10A1*, and *BAAT* central to bile acid metabolism; *PON1* and *CYP2C9* important for detoxification of xenobiotic compounds; *AFM*, *RBP4*, and *GC* that encode transport proteins; and *APOH* and *APOC1* involved in lipoprotein metabolism.

Conversely, tesamorelin was found to downregulate a hepatic stellate cell (HSC) gene signature predictive of poor HCC prognosis (NES = –1.63, FDR *q* value = 0.03) (Table 3 and Figure 5). Genes included within the leading edge of this gene set were involved in collagen biosynthesis and assembly (e.g., *COL5A2*, *LOXL2*), organization of the actin cytoskeleton (e.g., *TAGLN*, *TLN2*, *SDC3*), and growth factor signaling (e.g., *PDGFRB*, *HGF*, *PDGFRA*). Using a curated gene set, we further found that YAP/TAZ signaling, which is implicated in hepatic fibrogenesis and carcinogenesis (6), was downregulated among tesamorelin- versus placebo-treated participants over the 1-year study period (NES = –1.62, FDR *q* value = 0.03) (Table 3).

### Relationships of changes in hepatic gene expression with change in hepatic fat

For each differentially regulated gene set, we assessed whether change in gene expression related to change in hepatic fat fraction. In this regard, we compared 3 groups of participants: placebo-treated individuals, tesamorelin-treated individuals with less than 30% relative hepatic fat reduction, and tesamorelin-treated individuals with at least 30% relative hepatic fat reduction. Across these groups, consistent and significant trends were observed, demonstrating greater change in gene expression with a stepwise change in hepatic fat fraction from placebo- to tesamorelin-treated group with less than 30% fat reduction to tesamorelin-treated group with at least 30% fat reduction (i.e., greater change in gene expression with greater reduction in hepatic fat, Supplemental Table 2). No significant relationship was found between treatment status and the expression of de novo lipogenesis genes as assessed by Gene Ontology pathways.

### Relationships of changes in hepatic gene expression with change in fibrosis-related gene score and *IGF1* transcript levels

**Figure 6:**
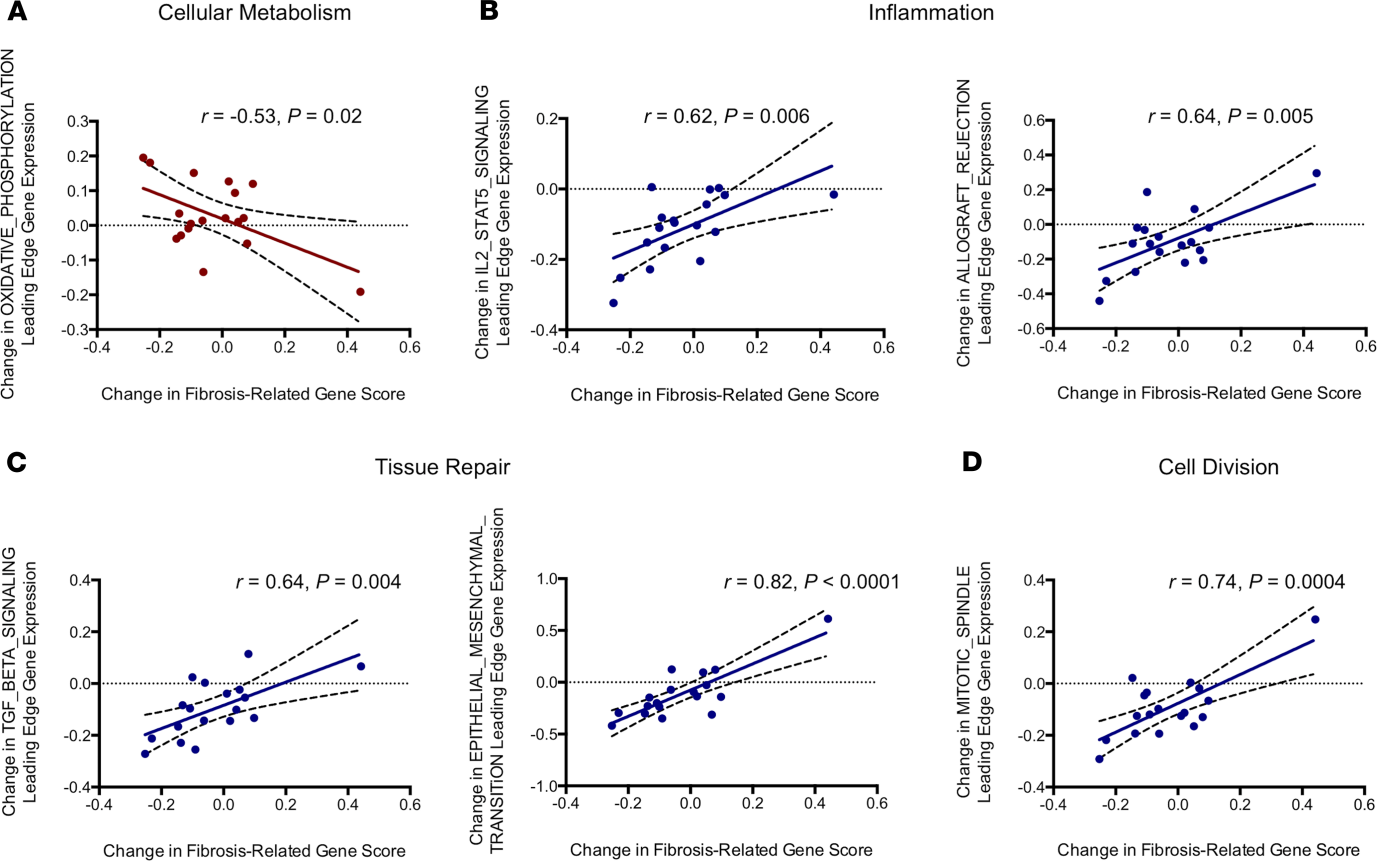
Changes in hepatic expression of hallmark gene sets were correlated with change in mean fibrosis-related gene score in tesamorelin-treated participants. (**A**) Within the tesamorelin group, a reduction in fibrosis-related gene score was associated with an increase in hepatic expression of oxidative phosphorylation genes. (**B**–**D**) In contrast, among tesamorelin-treated participants, change in fibrosis-related gene score was directly associated with changes in hepatic expression of gene sets pertaining to inflammation, tissue repair, and cell proliferation. For all graphs, axes reflect log_2_ fold change in mean leading edge gene expression. Linear regression lines with 95% confidence intervals are shown, with *r* and *P* value from Pearson correlation. *N* = 18 for all graphs. Graphs with red and blue dots correspond to pathways up- and downregulated by tesamorelin versus placebo, respectively.

Finally, among tesamorelin-treated participants, we assessed relationships between changes in hepatic expression of differentially regulated gene sets and change in fibrosis-related gene score based on the hepatic expression of 18 genes previously shown to correlate with fibrosis (7). Among our overall sample at baseline, we found a strong association between fibrosis-related gene score and histologic fibrosis stage (*P* = 0.0009; post-ANOVA test for linear trend *P* = 0.0001), which validated our use of this gene set as a proxy for hepatic fibrosis (Supplemental Figure 2). Of note, we demonstrated that hepatic upregulation of genes involved in oxidative phosphorylation and favorable HCC prognosis were associated with decreased fibrosis-related gene score. Moreover, hepatic downregulation of genes involved in inflammation, tissue repair, cell division, and unfavorable HCC prognosis also strongly correlated with decreased fibrosis-related gene score (Supplemental Table 3 and Figure 6).

Within the tesamorelin group, we also found that changes in hepatic *IGF1* transcript levels were associated with changes in pathways involved in tissue repair and carcinogenesis, including TGF_BETA_SIGNALING, UV_RESPONSE_DN, and YAP_TAZ_SIGNATURE. For each relationship, a greater rise in *IGF1* transcript levels was associated with more pronounced downregulation of these pathways (Supplemental Table 3, Supplemental Figure 3).

## Discussion

In HIV-associated NAFLD, we showed that tesamorelin, relative to placebo, increased hepatic expression of genes important for oxidative phosphorylation and decreased expression of genes involved in inflammation, tissue repair, and cell division. Furthermore, we found that treatment with tesamorelin led to reciprocal up- and downregulation of genes associated with favorable and poor HCC prognosis, respectively. Notably, these changes in hepatic gene expression correlated with improved fibrosis-related gene score among tesamorelin-treated participants. Taken together, our findings inform our knowledge of the biology of pulsatile GH action and provide a potential mechanistic basis for the observed clinical effects of tesamorelin on the liver.

In an unbiased analysis of the MSigDB hallmark gene sets, we found that tesamorelin led to hepatic upregulation of oxidative phosphorylation genes compared with placebo over 1 year. Furthermore, among tesamorelin-treated participants, enhanced expression of these genes related to oxidative phosphorylation was associated with decreased fibrosis-related gene score and degree of hepatic fat reduction using a clinically defined 30% stratification across treatment groups (8). Moreover, increases in oxidative phosphorylation were related to increased IGF-1 transcription, providing evidence linking augmented GH signaling to increased oxidative phosphorylation. Mitochondria play a key role in fatty acid catabolism, and dysfunction in this key organelle has been implicated as a key feature in NAFLD pathogenesis (9). In a NASH rat model, declines in hepatic oxidative phosphorylation efficiency, electron transport chain enzyme activities, and mitochondrial transmembrane potential were seen as hepatic steatosis progressed (10). Similarly, liver biopsy specimens obtained from patients with NASH were found to have reduced maximal mitochondrial respiration and blunted expression of transcription factors regulating mitochondrial biogenesis and the electron transport chain versus controls (11). Mitochondrial impairment may promote hepatic fat accumulation and generation of toxic lipid metabolites, increasing oxidative stress, cell death, inflammation, and fibrosis, which are key events in NAFLD progression (9). Strategies to restore oxidative phosphorylation may be useful to ameliorate the clinical course of NAFLD progression (12).

Our finding that tesamorelin upregulated expression of hepatic oxidative phosphorylation genes is supported by prior work showing that GH axis augmentation has beneficial mitochondrial effects. In a small study of healthy men and women, short-term infusion of GH acutely elevated skeletal muscle mitochondrial oxidative capacity, heightened the abundance of muscle mRNAs encoding mitochondrial proteins, and shifted whole-body substrate utilization toward fat oxidation (13). Similarly, in a rat model of cirrhosis, treatment with IGF-1 was demonstrated to increase mitochondrial membrane potential and ATP synthase activity and to reduce intramitochondrial free radical production, caspase activation, and apoptosis (14). Last, in obese adults treated with tesamorelin, we previously found an association between increased serum IGF-1 and accelerated ATP-dependent phosphocreatine recovery rate in skeletal muscle following exercise (15). Overall, improved mitochondrial function in response to enhanced GH secretion may be a key strategy whereby tesamorelin attenuates NAFLD severity in HIV.

Compared with placebo, we also showed that tesamorelin led to robust downregulation of key inflammatory gene pathways involved in the innate and adaptive immune response. Notable among the gene sets differentially regulated between groups were those involved in tumor necrosis factor–α (TNF-α) and interleukin-6 (IL-6) signaling. Specifically, studies have shown that hepatic expression of these cytokines was elevated in patients with NASH in proportion to the degree of inflammation (16, 17).

The effects of tesamorelin on multiple inflammatory pathways suggest that augmenting endogenous GH may be effective in reducing hepatic inflammation in HIV-associated NAFLD. Consistent with these findings, prior studies have shown that GH deficiency is a systemic inflammatory state that is attenuated by GH axis augmentation (18, 19). Additionally, in a mouse model, liver-specific ablation of the GH receptor led to hepatic steatosis with increased macrophage infiltration and enhanced hepatic expression of cytokines, including TNF-α and IL-6 (20). In our recent clinical trial, change in histologic inflammation was not found to significantly differ between treatment and placebo groups, though sample size was limited. Nonetheless, tesamorelin-treated individuals with higher histologic inflammation at baseline exhibited a greater decline in inflammatory activity over 1 year (3). In addition, compared with placebo, tesamorelin was demonstrated to reduce systemic immune activation as measured by circulating C-reactive protein levels (3). As such, our gene expression data expand upon the clinical signal that we observed.

In the current study, we also found that decreased hepatic expression of inflammatory gene sets was associated with a reduction in fibrosis-related gene score among tesamorelin-treated participants. Given that NASH is an important precursor to fibrosis, reducing hepatic inflammation may provide a mechanistic basis whereby tesamorelin slows fibrosis progression in HIV-associated NAFLD. Evaluation of specific leading edge genes in the inflammatory gene sets differentially regulated by tesamorelin versus placebo suggests potential pathways that may mediate the fibrogenic response. As 2 examples, leading edge genes C-C motif chemokine ligands 5 (*CCL5*) and 20 (*CCL20*) are highly expressed among patients with NAFLD in proportion to the severity of histologic disease (21, 22). In vitro treatment of HSCs with CCL5 or CCL20 has been shown to directly induce fibrogenesis (21, 22). Moreover, in a recent phase IIb trial of NASH, antagonism of CCL5 receptors with cenicriviroc reduced hepatic fibrosis without worsening steatohepatitis (23). Taken together, downregulation of inflammatory genes that dually promote fibrogenesis may contribute to the relationship that we observed between changes in inflammatory pathways and fibrosis gene signature among tesamorelin-treated participants.

Importantly, we additionally found that tesamorelin led to hepatic downregulation of gene pathways involved in tissue repair, including those related to apoptosis, EMT, and TGF-β signaling, compared with placebo. Moreover, these changes in gene expression directly correlated with change in fibrosis-related gene score within the tesamorelin group. While tissue repair responses enable liver regeneration following an acute insult, ongoing activation of these pathways in the setting of chronic injury may lead to hepatic fibrosis (24). As an example, high rates of hepatocyte apoptosis, as has been described in patients with NAFLD, may trigger fibrogenesis (25). Similarly, TGF-β signaling is the most prominent pathway driving hepatic fibrogenesis (26). In this regard, TGF-β signaling results in activation of HSCs, transdifferentiation of epithelial cells to myofibroblasts, and enhanced production of ECM proteins (26, 27). Downregulation of the tissue repair response by tesamorelin may constitute an important process by which this agent slows the clinical progression of liver disease.

Our findings that tesamorelin reduced hepatic expression of genes involved in tissue repair support a role for augmented GH secretion and IGF-1 in blunting fibrogenesis. In this regard, data in the current study demonstrate significant inverse relationships between changes in *IGF1* transcript levels and key tissue repair pathways. Relatedly, in both NASH and cirrhotic animal models, augmentation of hepatic IGF-1 signaling has been shown to result in fibrosis regression (28, 29). In one such study, IGF-1 treatment led to hepatic upregulation of metalloproteinases responsible for ECM degradation, as well as downregulation of the profibrogenic mediator *Tgfb* and metalloproteinase inhibitors *Timp1* and *Timp2* (29). Of note, the changes in gene expression in this prior report resembled those that we observed with GHRH analog therapy, which we now extend for the first time to our knowledge to humans. IGF-1 has been demonstrated to decrease apoptosis in multiple cell lines (30) and to induce senescence of HSCs in a p53-dependent manner (28), which may underlie these phenotypic changes.

Tesamorelin downregulated hepatic genes involved in cell division as compared with placebo. Furthermore, reduced expression of these genes among tesamorelin-treated individuals was associated with a decrease in fibrosis-related gene scores. Hepatocyte proliferation has been recognized as a critical step underlying NAFLD pathogenesis even early in the course of the disease (31). In this regard, liver specimens from patients and animal models with NAFLD have consistently shown elevations in markers of cell proliferation, including Ki-67, E2F1, cyclin-dependent kinase 4, and gank (31–33). Importantly, such factors have been implicated in the development of hepatic steatosis and fibrosis. As an example, in *db/db* leptin-deficient mice, *E2f1* knockout was found to prevent hepatic steatosis through crosstalk with key metabolic pathways (34). Furthermore, in a mouse model of cirrhosis, E2F1 deficiency was demonstrated to protect against liver fibrosis and associated hepatic dysfunction (33). Beyond accelerating NAFLD progression, high rates of cell division may predispose to HCC. Notably, the minichromosome maintenance genes *MCM2* and *MCM6*, which were both downregulated in tesamorelin-treated participants, were shown to be elevated in HCC in association with poor survival (35).

Tesamorelin reduced fibrosis progression in our clinical trial, an important factor associated with the development of HCC. Although assessment of HCC outcomes was beyond the scope of our trial, we used curated gene sets in this analysis to assess potential relationship to HCC risk. We found that tesamorelin upregulated genes associated with a favorable HCC prognosis and downregulated genes associated with poor HCC prognosis. Tesamorelin also downregulated the YAP/TAZ signaling pathway, which is a key driver of fibrosis and cancer (6). This favorable modulation of cancer-related gene sets parallels the widespread improvement in metabolic, inflammatory, fibrogenic, and proliferative pathways that we observed in the current analysis. Importantly, these changes also run counter to theoretical concerns that increased GH/IGF-1 signaling may exacerbate cancer risk, which have been raised in some contexts. In this regard, it is important to consider that GHRH analog therapy, unlike GH, augments physiologic GH pulsatility with gains in IGF-1 that remain within the normal range.

To our knowledge, this is the first study in humans to investigate the mechanistic underpinnings for a NAFLD therapy using a whole-transcriptome approach. Additionally, study participants comprised a modern cohort of PLWH, and the results derive from a 1-year randomized controlled trial with a placebo comparator. Given that the HIV population is at high risk of NAFLD and increased fibrosis progression rates (1, 2, 36), there is a critical need for dedicated studies among this patient group. Our findings also may yield insights for other populations with NAFLD and thus provide a strong rationale for additional studies. Limitations of our analysis include its relatively small sample size. Moreover, although the sample was a subset of our larger study cohort, we do not believe there were any factors systematically contributing to selection into the subset, as supported by the overall similarities between this study cohort and the entire cohort. Because we have tissue from only 2 time points, we cannot determine whether changes in gene expression are a direct effect of tesamorelin or are mediated by other biological effects indirectly resulting from tesamorelin. Thus, although we saw significant correlations between increased IGF-1 transcription and changes in gene expression, further studies are needed to assess whether these effects are directly related to augmented GH signaling. Though we did not see effects over time of tesamorelin to downregulate de novo lipogenesis gene pathways, our assessments were made in the fasting state, when lipogenesis is low. Additionally, GH axis augmentation may result in modulation of pathways at the protein translational or posttranslational level that would evade detection at a transcriptome level. Relatedly, a change in gene expression does not necessarily correspond to changes in tissue function.

In summary, among individuals with HIV-associated NAFLD, GH axis augmentation with tesamorelin led to changes in hepatic gene expression that reflect an overall return toward liver health. We found that tesamorelin upregulated oxidative phosphorylation genes and downregulated genes involved in inflammation, tissue repair, and cell turnover compared with placebo. Further, tesamorelin shifted hepatic gene expression toward a profile associated with a favorable HCC prognosis. By expanding our knowledge of the effects of GH axis augmentation on hepatic biology, our findings raise the possibility of novel clinical benefits of this strategy in the treatment of NASH that should be evaluated in future large-scale efforts.

## Methods

### Study design.

We previously conducted a randomized, double-blind trial in which individuals with HIV-associated NAFLD were assigned to receive the GHRH analog tesamorelin 2 mg daily or identical placebo for 12 months (3). The current analysis leveraged liver biopsy specimens from this recent trial to identify gene pathways that were differentially modulated by treatment and to investigate associations of changes in gene expression with changes in clinical outcome among tesamorelin-treated individuals. These findings have not been previously reported.

We enrolled 61 men and women 18–70 years old who had documented HIV infection as well as hepatic steatosis as defined by liver fat fraction at least 5% on ^1^H–magnetic resonance spectroscopy (^1^H-MRS). Participants were required to have been on stable ART for at least 3 months with CD4^+^ T cell count greater than 100 cells/mm^3^ and HIV viral load less than 400 copies/mL. Exclusion criteria included excess alcohol use (>20 g daily for women or >30 g daily for men), active hepatitis B or C as previously described (3), other known hepatic disease, cirrhosis, and inadequately controlled diabetes mellitus (HbA1c ≥ 7%). Participants were enrolled at the Massachusetts General Hospital (MGH, Boston, Massachusetts) and the NIH (Bethesda, Maryland) between August 20, 2015, and January 16, 2019.

### Study procedures.

All study procedures were conducted in a fasting state. Hepatic ^1^H-MRS was performed for measurement of hepatic fat fraction. Baseline evaluation also included an ultrasound-guided percutaneous liver biopsy yielding 2 cores, which was completed on all participants except for those with a contraindication (e.g., anticoagulation). The first core was fixed in formalin and subsequently underwent histopathologic review by a single expert pathologist blinded to treatment. Histologic scoring, including NAFLD Activity Score and fibrosis stage, was performed according to the Nonalcoholic Steatohepatitis Clinical Research Network scoring system (37). The second core was placed in an RNA stabilization reagent (RNAlater, QIAGEN) and stored at –80°C for gene expression analysis. Both ^1^H-MRS and liver biopsy were repeated at 12 months following randomization.

### cDNA library construction.

Following extraction from liver tissue using RNeasy Plus Mini Kit (QIAGEN), total RNA was quantified using the Quant-iT RiboGreen RNA Assay Kit (Thermo Fisher Scientific) and normalized to 5 ng/μL. Following plating, 2 μL of External RNA Controls Consortium controls (using a 1:1000 dilution) and a k562 control were spiked into each sample. A 200 ng aliquot of each sample was taken for library preparation, using Illumina TruSeq Stranded mRNA Sample Preparation Kit. Oligo-dT beads were used to select mRNA from the total RNA sample, followed by heat fragmentation and cDNA synthesis from the RNA template. The resultant 400 bp cDNA underwent dual-indexed library preparation: “A” base addition, adapter ligation using P7 adapters, and PCR enrichment using P5 adapters. After enrichment the libraries were quantified using Quant-iT PicoGreen (Thermo Fisher Scientific, 1:200 dilution).

### Illumina sequencing.

Pooled libraries were normalized to 2 nM and denatured using 0.1N NaOH before sequencing. Flow cell cluster amplification and sequencing were performed according to the manufacturer’s protocols using the NovaSeq S2 (Illumina) to produce 101 bp paired-end reads with 8-base index barcodes. Data were analyzed using the Broad Picard Pipeline, which includes demultiplexing and data aggregation.

### Alignment and quality control.

All samples were analyzed using the bcbio-nextgen RNA-Seq analysis pipeline (https://bcbionextgen.readthedocs.org/en/latest/). BAM files (converted back to FASTQ read files) were examined for quality issues using FastQC (http://www.bioinformatics.babraham.ac.uk/projects/fastqc/) to ensure library generation and sequencing were suitable for further analysis. Reads were aligned to UCSC build hg38 of the human genome (*Homo sapiens*), augmented with transcript information from Ensembl release 94 using hisat2 (38). Alignments were analyzed for evenness of coverage, rRNA content, genomic context (for example, alignments in known transcripts and introns), complexity, and other quality checks using a combination of FastQC, Qualimap (39), MultiQC (https://github.com/ewels/MultiQC), and custom tools. Counts of reads aligning to known genes were generated by featureCounts (40) and used as input for principal components analysis and hierarchical clustering to identify possible outliers. Additional patterns of gene expression were visualized using the DEGReport Bioconductor package (41).

A total of 19 participants randomized to tesamorelin and 24 participants randomized to placebo had paired liver specimens available for gene sequencing. Four participants, 1 assigned to tesamorelin and 3 to placebo, were excluded from subsequent analyses because of poor RNA samples, resulting in a total of 18 participants randomized to tesamorelin and 21 randomized to placebo with RNA-Seq data included in our analysis. A comparison between characteristics of the participants included in this study and the entire study cohort is shown in Supplemental Table 1. The RNA-Seq data were submitted to the Gene Expression Omnibus repository at the National Center for Biotechnology Information (accession number GSE150026).

### Gene set enrichment analysis.

To identify pathways differentially modulated from pre- to posttreatment time points between tesamorelin- and placebo-treated participants, GSEA was performed using the desktop module from the Broad Institute (www.broadinstitute.org/gsea/). First, transcripts per million measurements per isoform were generated by quasialignment using Salmon (42). Differential expression at the gene level was called with DESeq2 (43) using counts per gene estimated from the Salmon quasialignments by tximport (44). To determine changes in gene expression over time between treatment groups, we used a repeated-measures model that controlled for subject-specific effects, such as sex, and used an interaction term of treatment × time point.

Next, transcripts were ranked between tesamorelin- and placebo-treated participants, using the DESeq2 test statistic (Wald’s test). Transcripts with greater upregulation from baseline in tesamorelin-treated participants relative to placebo were ranked at the top of the list, and those with greater downregulation in tesamorelin-treated participants relative to placebo at the bottom of the list. GSEA was performed on the ranked transcript list using 1000 gene set permutations and random seeding. GSEA leading edge genes are the subset of genes in a significantly enriched gene set that account for the enrichment signal and were used for subsequent quantification of pathway gene expression. Heatmaps of leading edge gene log_2_ fold change expression levels from pretreatment to posttreatment time points were generated with pheatmap (45). Transcripts were organized on the *y* axis by unsupervised hierarchical clustering (Euclidean distance, complete linkage), and samples were manually organized by treatment on the *x* axis. Gene sets with FDR < 0.05 were considered enriched.

Gene sets used included the MSigDB hallmark gene set collection (4) and custom gene sets. We examined a custom set of genes associated with a favorable prognosis in HCC. To develop this set, we searched the Human Protein Atlas for genes expressed in hepatic tissue that were associated with a favorable prognosis in HCC (46). The resulting gene list was filtered to exclude genes previously described in a prognostic liver signature (47) to produce our HCC_FAVORABLE_PROGNOSIS gene set. Furthermore, we derived a gene set predictive of poor HCC prognosis from a subset of HSC signature genes that have been previously correlated with poor HCC outcome (HCC_POOR_PROGNOSIS) (48). Relatedly, to study a key pathway involved in carcinogenesis, we leveraged a published set of YAP/TAZ signature genes that had been characterized through in silico meta-analyses of Hippo signaling modules in cancer (YAP_TAZ_SIGNATURE) (49).

### Correlation analyses.

DESeq2 variance stabilizing–transformed data were used to calculate the log_2_ fold change in gene expression between pretreatment and posttreatment time points. Mean values for leading edge gene log_2_ fold change were calculated for each gene set on each subject.

Similarly, for each subject, we calculated a fibrosis-related gene score as the mean log_2_ fold change for a set of 18 genes (FIBROSIS_SIGNATURE) that was previously identified to be predictive of fibrosis stage in an analysis of patients with NAFLD (7).

### Statistics.

Differential gene expression between tesamorelin- and placebo-treated patients was calculated using negative binomial generalized linear models (DESeq2). We assessed the relationship between mean fibrosis-related gene score and histologic fibrosis stage among our subjects at baseline using a 1-way ANOVA including a test for linear trend (GraphPad Prism). Pearson correlation was then used to compare the change in mean leading edge gene transcript abundance to the change in mean transcript abundance of the FIBROSIS_SIGNATURE set among the tesamorelin group. Additional comparisons were made of mean leading edge gene fold change expression values with fold change in *IGF1* expression. In addition, changes in mean leading edge gene expression were assessed in relationship to responsiveness to tesamorelin as defined by a clinically significant reduction in hepatic fat fraction that has been previously established (8). In this regard, we compared changes in gene expression among 3 groups: placebo-treated individuals, tesamorelin-treated individuals with less than 30% relative hepatic fat reduction, and tesamorelin-treated individuals with at least 30% relative hepatic fat reduction, using 1-way ANOVA for overall effects and linear trend analysis. Statistical tests with *P* < 0.05 were considered statistically significant. We used the Benjamini-Hochberg procedure when correcting for multiple tests.

### Study approval.

Informed consent in writing was obtained from each participant. The institutional review boards at MGH and the NIH approved this study.

## Author contributions

LTF contributed to the conduct of the study, conception and execution of the analyses presented, and preparation of the manuscript. JMB contributed to the conception and execution of the analyses presented and preparation of the manuscript. GA contributed to the conception and execution of the analyses presented and preparation of the manuscript. SJHS contributed to the conception and execution of the analyses presented and preparation of the manuscript. MNF contributed to the conduct of the study and editing of the manuscript. JP contributed to the conduct of the study and editing of the manuscript. IZ contributed to the conduct of the study and editing of the manuscript. CSP contributed to the conduct of the study and editing of the manuscript. KEC contributed to the conduct of the study and editing of the manuscript. MT contributed to the conduct of the study and editing of the manuscript. DEK contributed to the conduct of the study, histopathologic review, and editing of the manuscript. CMH contributed to the design and conduct of the study, the conception of the analyses presented, and editing of the manuscript. TLS contributed to the design and conduct of the study and preparation and editing of the manuscript. RTC contributed to the conception and execution of the analyses presented and preparation of the manuscript. SKG contributed to the design and conduct of the study, conception and execution of the analyses presented, and preparation and editing of the manuscript.

## Supplementary Material

Supplemental data

## Figures and Tables

**Table 1:**
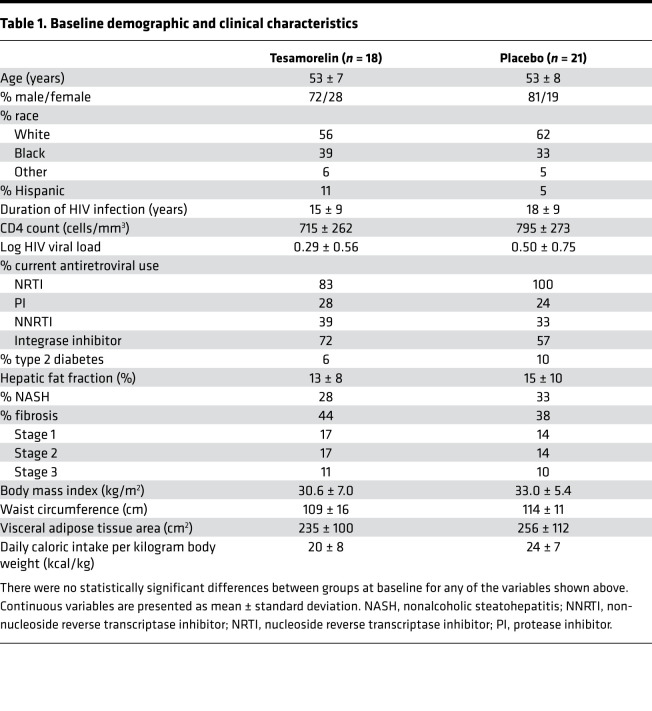
Baseline demographic and clinical characteristics

**Table 2:**
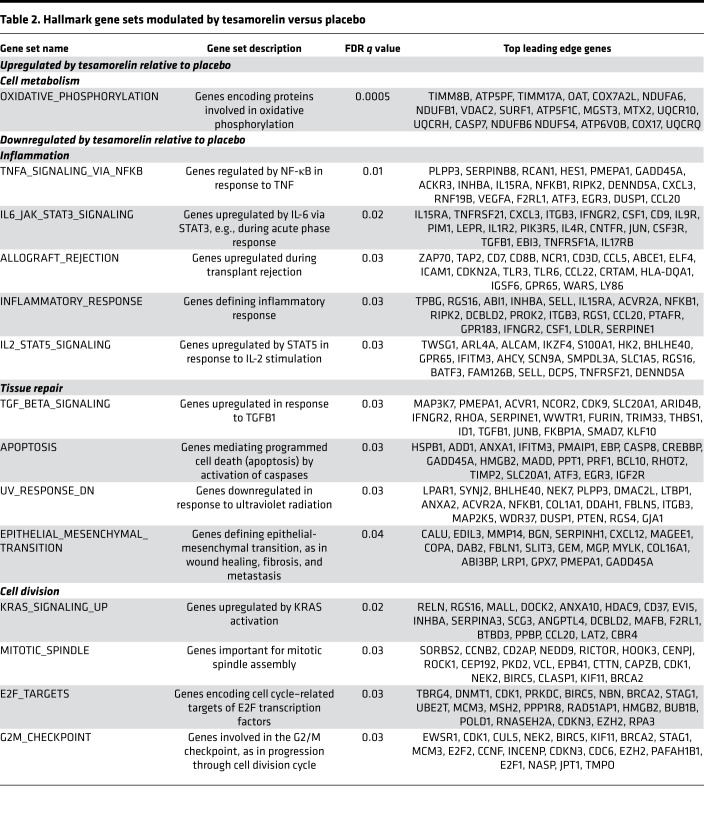
Hallmark gene sets modulated by tesamorelin versus placebo

**Table 3:**
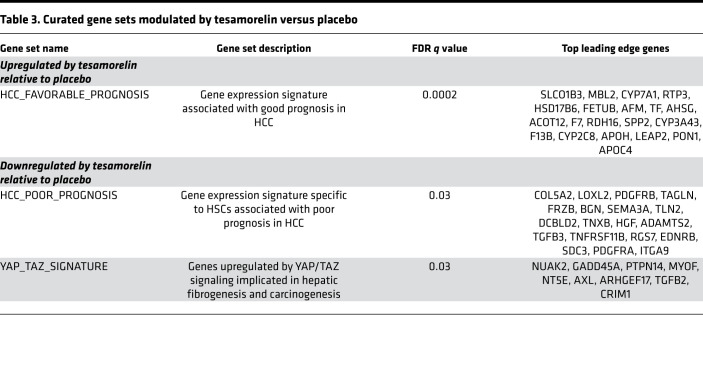
Curated gene sets modulated by tesamorelin versus placebo

## Version 1 07/23/2020

In-Press Preview

## Version 2 08/20/2020

Electronic publication
