# Supplementary material for: Effects of tesamorelin on hepatic transcriptomic signatures in HIV-associated NAFLD
Source: JCI Insight. 2020 Aug 20;5(16):e140134. doi: 10.1172/jci.insight.140134 (PMC7455119; doi:10.1172/jci.insight.140134)
Supplement: Supplemental data [file jciinsight-5-140134-s131.pdf]

Supplementary Table 1: Comparison Between Entire Study Cohort and RNA Sequencing Cohort

	RNA Sequencing Cohort (N=39)		Entire Study Cohort (N=61)	
	**Tesamorelin** (n = 18)	**Placebo** (n = 21)	**Tesamorelin** (n = 31)	**Placebo** (n = 30)
Age (years)	53 ± 7	53 ± 8	52 ± 8	54 ± 7
% Male	72	81	77	80
% Race				
White	56	62	68	63
Black	39	33	26	33
Other	6	5	7	3
% Hispanic	11	5	19	10
Duration of HIV infection (years)	15 ± 9	18 ± 9	16 ± 9	18 ± 8
CD4 count (cells/mm^3)	715 ± 262	795 ± 273	733 ± 290	798 ± 260
log HIV viral load	0.29 ± 0.56	0.50 ± 0.75	0.34 ± 0.59	0.50 ± 0.74
% Current Antiretroviral Use				
NRTI	83	100	87	97
PI	28	24	29	20
NNRTI	39	33	39	37
Integrase Inhibitor	72	57	68	60
% Type 2 Diabetes	6	10	13	13
Hepatic Fat Fraction (%)	13 ± 8	15 ± 10	13 ± 8	15 ± 10
% NASH	28	33	35	31
% Fibrosis	44	38	48	38
Stage 1	17	14	14	17
Stage 2	17	14	21	14
Stage 3	11	10	14	7
Body mass index (kg/m^2)	30.6 ± 7.0	33.0 ± 5.4	30.1 ± 6.0	32.9 ± 6.2
Waist circumference (cm)	109 ± 16	114 ± 11	107 ± 15	114 ± 12
Visceral adipose tissue area (cm^2)	235 ± 100	256 ± 112	232 ± 91	250 ± 104
Daily caloric intake per kg body weight (kcal/kg)	20 ± 8	24 ± 7	23 ± 9	23 ± 7

Supplementary Table 2: Relationships of Changes in Hepatic Gene Expression with Change in Hepatic Fat

	**P-Value for Overall ANOVA***	**P-Value for Linear Trend***	**Slope**	**95% CI of Slope**
OXIDATIVE_PHOSPHORYLATION	0.04	0.02	0.05414	0.01054 to 0.09774
HCC_FAVORABLE_PROGNOSIS	0.05	0.03	0.07936	0.007485 to 0.1512
TNFA_SIGNALING_VIA_NFKB	0.0004	0.0003	-0.1083	-0.1627 to -0.05382
IL6_JAK_STAT3_SIGNALING	0.0004	<0.0001	-0.1176	-0.1714 to -0.06376
ALLOGRAFT_REJECTION	0.02	0.008	-0.08164	-0.1404 to -0.02288
INFLAMMATORY_RESPONSE	0.0002	<0.0001	-0.1084	-0.1572 to -0.05961
IL2_STAT5_SIGNALING	<0.0001	<0.0001	-0.1091	-0.1487 to -0.06962
TGF_BETA_SIGNALING	0.005	0.003	-0.09041	-0.1479 to -0.03290
APOPTOSIS	0.001	0.0003	-0.1131	-0.1698 to -0.05635
UV_RESPONSE_DN	<0.0001	<0.0001	-0.09884	-0.1420 to -0.05568
EPITHELIAL_MESENCHYMAL_TRANSITION	0.06	0.03	-0.1017	-0.1898 to 0.01363
KRAS_SIGNALING_UP	0.001	0.0004	-0.1041	-0.1586 to -0.04956
MITOTIC_SPINDLE	0.0004	0.001	-0.08051	-0.1264 to -0.03458
E2F_TARGETS	0.0002	0.0007	-0.09821	-0.1517 to -0.04474
G2M_CHECKPOINT	<0.0001	0.0006	-0.09628	-0.1484 to -0.04419
HCC_POOR_PROGNOSIS	0.3	0.1	-0.07516	-0.1783 to 0.02797
YAP_TAZ_SIGNATURE	0.003	0.0009	-0.1298	-0.2023 to -0.05733

Statistical comparison of changes in expression of gene sets with changes in hepatic fat fraction, across three participant groups: placebo, tesamorelin treated with <30% relative change in hepatic fat fraction, and tesamorelin treated with ≥30% relative change in hepatic fat fraction. The slope represents change from no treatment (placebo) to treatment with lower change in hepatic fat (<30%) to treatment with greater change in hepatic fat (≥30%).

Supplementary Table 3: Correlations of Changes in Differentially Regulated Gene Sets with Change in *IGF1* Transcript Level and Fibrosis-Related Gene Score Among Tesamorelin-Treated Participants

	**Change in *IGF1* Transcript Level**	**Change in Fibrosis-Related Gene Score**
OXIDATIVE_PHOSPHORYLATION	r = 0.42, *P* = 0.08	r = -0.53, ***P* = 0.02**
HCC_FAVORABLE_PROGNOSIS	r = 0.62, ***P* = 0.006**	r = -0.79, ***P* < 0.0001**
TNFA_SIGNALING_VIA_NFKB	r = -0.39, *P* = 0.11	r = 0.50, ***P* = 0.03**
IL6_JAK_STAT3_SIGNALING	r = -0.27, *P* = 0.28	r = 0.27, *P* = 0.28
ALLOGRAFT_REJECTION	r = -0.34, *P* = 0.16	r = 0.64, ***P* = 0.005**
INFLAMMATORY_RESPONSE	r = -0.31, *P* = 0.21	r = 0.46, *P* = 0.06
IL2_STAT5_SIGNALING	r = -0.34, *P* = 0.16	r = 0.62, ***P* = 0.006**
TGF_BETA_SIGNALING	r = -0.49, ***P* = 0.04**	r = 0.64, ***P* = 0.004**
APOPTOSIS	r = -0.38, *P* = 0.12	r = 0.74, ***P* = 0.0005**
UV_RESPONSE_DN	r = -0.53, ***P* = 0.02**	r = 0.60, ***P* = 0.009**
EPITHELIAL_MESENCHYMAL_TRANSITION	r = -0.33 *P* = 0.18	r = 0.82, ***P* < 0.0001**
KRAS_SIGNALING_UP	r = -0.35, *P* = 0.16	r = 0.77, ***P* = 0.0002**
MITOTIC_SPINDLE	r = -0.54, ***P* = 0.02**	r = 0.74, ***P* = 0.0004**
E2F_TARGETS	r = -0.01, *P* = 0.97	r = 0.54, ***P* = 0.02**
G2M_CHECKPOINT	r = -0.06, *P* = 0.83	r = 0.52, ***P* = 0.03**
HCC_POOR_PROGNOSIS	r = -0.41, *P* = 0.09	r = 0.84, ***P* < 0.0001**
YAP_TAZ_SIGNATURE	r = -0.57, ***P* = 0.01**	r = 0.52, ***P* = 0.03**

Supplementary Figure 1

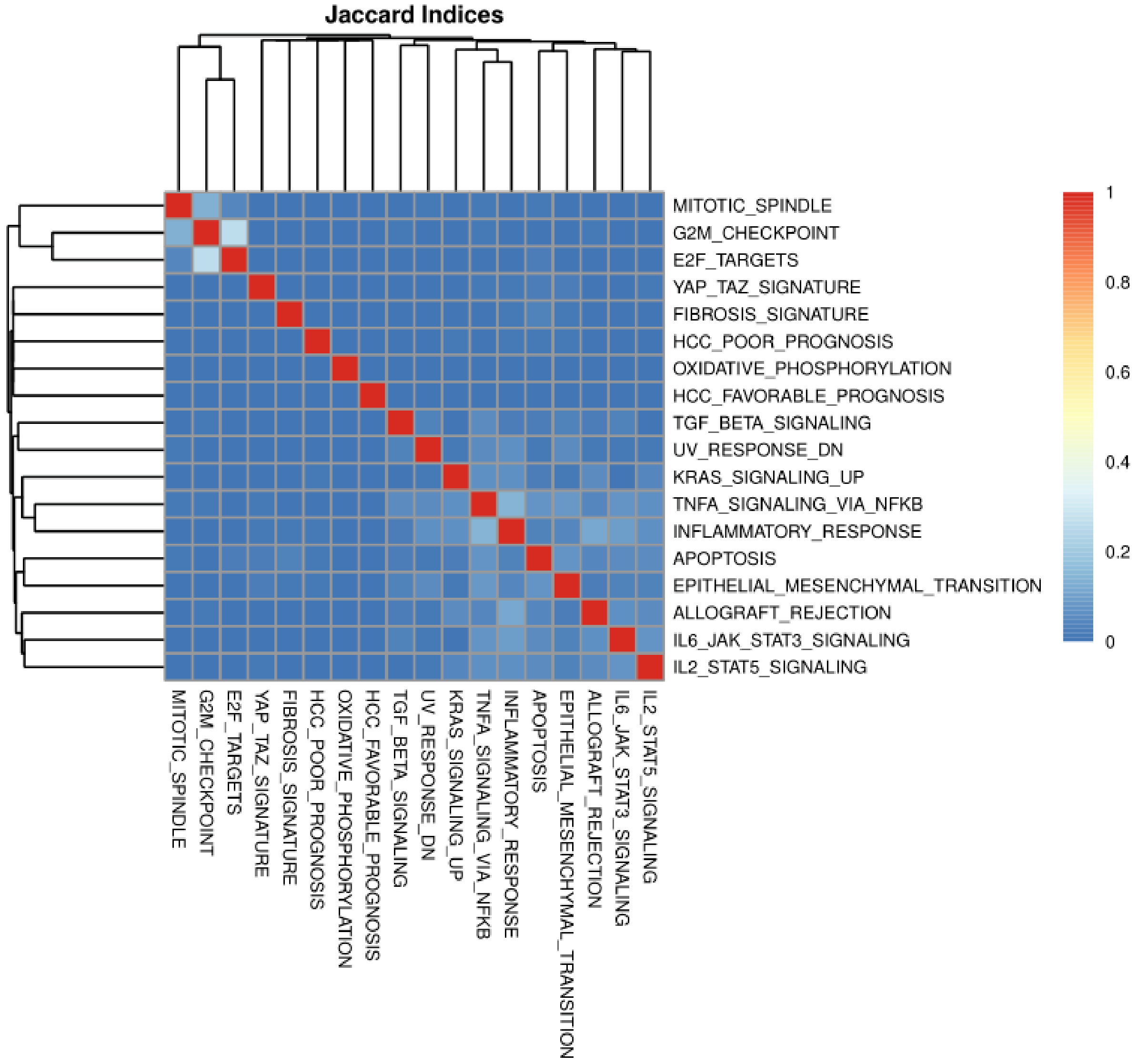

Jaccard Plot for Differentially Regulated Gene Sets. A Jaccard plot depicts correlations between gene sets that were differentially modulated by treatment status. Notably, there was minimal overlap between gene sets, which reinforces the independence of our findings.

Supplementary Figure 2

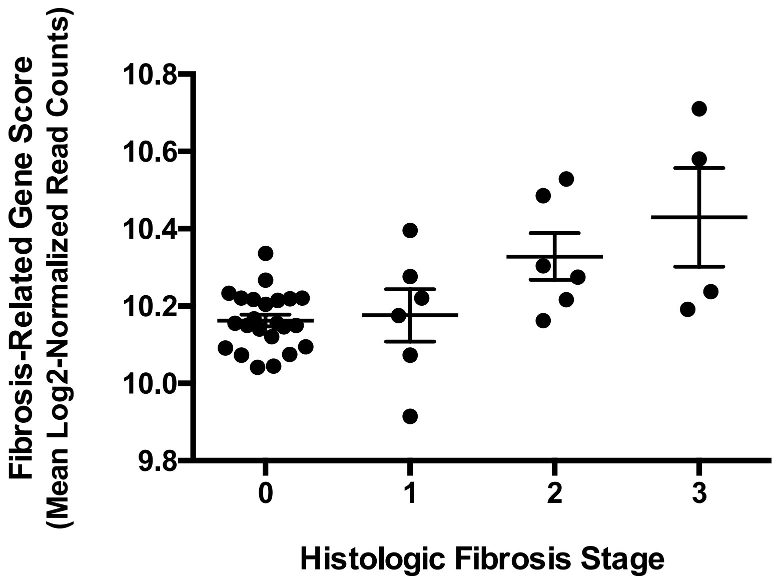

Fibrosis-Related Gene Score Was Associated with Histologic Fibrosis Stage. In our overall sample at baseline, we found a strong correlation between histologic fibrosis stage and fibrosis-related gene score, which was derived from a previously published gene set (Hoang et al., *Sci Rep*, 2019; 9(1):12541). P-value for ANOVA P = 0.0009; post-ANOVA test for trend P-value 0.0001. This finding validated the use of fibrosis-related gene score in our analysis as a proxy for liver histology.

Supplementary Figure 3

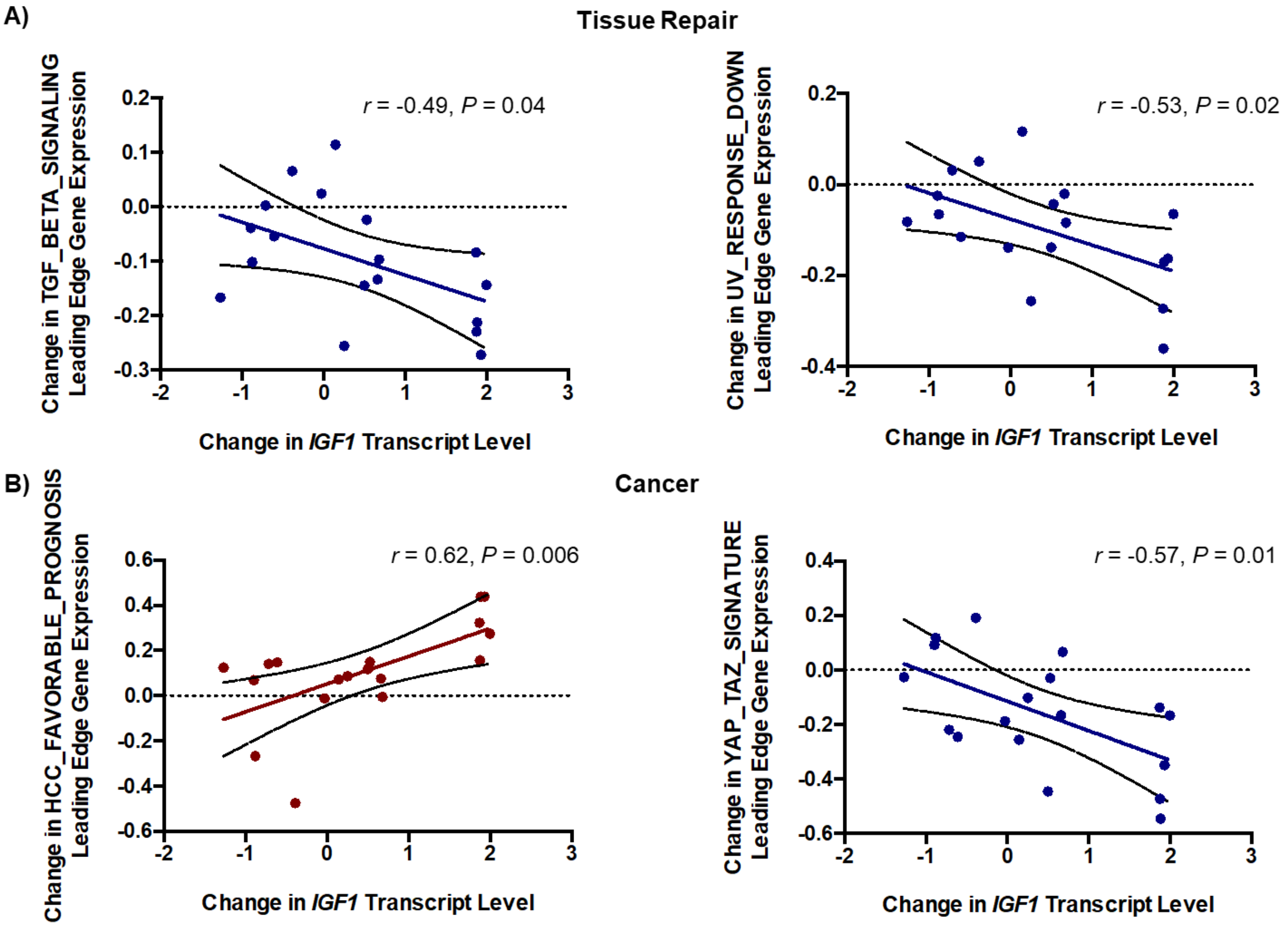

Change in *IGF1* Transcript Level Was Associated with Changes in Hepatic Expression of Tissue Repair and Cancer-Related Gene Sets in Tesamorelin-Treated Participants. A) Within the tesamorelin group, a greater augmentation in hepatic *IGF1* transcript level was associated with a more marked decline in tissue repair gene pathways. **B)** Likewise, a greater rise in hepatic *IGF1* transcript level was correlated with more favorable changes in the expression of hepatocellular carcinoma gene sets. For all graphs, axes reflect $\log_2$-fold change in gene expression. Linear regression lines with 95% confidence intervals are shown. Graphs with red and blue dots correspond to pathways up- and downregulated by tesamorelin versus placebo, respectively.
